# Fever and Hyponatremia Unmasking Brugada Pattern Electrocardiogram in a Patient With SARS-CoV-2 Infection

**DOI:** 10.7759/cureus.18578

**Published:** 2021-10-07

**Authors:** Sarah Ayad, Ramez Alyacoub, Kirolos Gergis, Muhammad Atif Masood Noori, Sherif Elkattawy, Basel Abdelazeem, Raja Pullatt

**Affiliations:** 1 Internal Medicine, Rutgers-New Jersey Medical School/Trinitas Regional Medical Center, Elizabeth, USA; 2 Internal Medicine, McLaren Health Care, Flint, USA; 3 Internal Medicine, Dow Medical College, Karachi, PAK; 4 Internal Medicine, Trinitas Regional Medical Center, Elizabeth, USA; 5 Internal Medicine, McLaren Health Care, Michigan State University, Flint, USA; 6 Cardiology, Internal Medicine, Rutgers-New Jersey Medical School/Trinitas Regional Medical Center, Elizabeth, USA

**Keywords:** electrocardiogram, hyponatremia, fever, brugada syndrome, covid-19, sars-cov-2

## Abstract

Brugada syndrome is an autosomal dominant genetic disorder that primarily affects myocardial sodium channels and has been associated with an increased risk of ventricular tachyarrhythmias and sudden cardiac death. Here, we report a case of a 58-year-old Hispanic male with a history significant for prior pulmonary tuberculosis infection who presented with pleuritic left-sided chest pain associated with body aches, productive cough, fevers, and chills and was found to be positive for SARS-CoV-2 by real-time reverse-transcription-polymerase chain reaction (rRT-PCR). Electrocardiogram (ECG, EKG) on presentation demonstrated a coved ST-segment elevation in V1-V2, suggesting Brugada pattern type 1 without evidence of ischemic changes. EKG changes normalized once fever and hyponatremia improved.

## Introduction

Brugada syndrome is an autosomal dominant genetic disorder that affects the myocardial sodium channel triggering abnormal electrical activity in the heart [1,2]. Patients having typical EKG patterns who have experienced sudden cardiac death, or sustained ventricular arrhythmia is said to have Brugada syndrome. Brugada ECG pattern is characterized by pseudo right bundle branch block and persistent ST-segment elevation in leads V1-V3. Two distinct patterns of ST-elevation have been found; in type 1, the elevated ST-segment (>2 mm) descends with an upward convexity to an inverted T-wave, referred to as the “coved type” Brugada pattern. Type 2 (Saddleback type) the elevated ST segment descends toward the baseline, then rises again to an upright or biphasic T-wave [1,2]. Fever and electrolyte abnormalities are the most important triggers that contribute to EKG and clinical manifestations of Brugada syndrome. Management includes lifestyle modifications to avoid triggers, medications, and prevention of sudden cardiac death by implantable cardioverter-defibrillator [1,2]. We report a case of Brugada pattern induced by hyponatremia and fever associated with COVID-19.

## Case presentation

A 58-year-old Hispanic male with a history significant only for pulmonary tuberculosis diagnosed over a decade ago, who presented to the emergency department (ED) complaining of left-sided pleuritic chest pain that was localized, and sharp. This was associated with body aches, productive cough, fevers, and chills. He reported exposure to COVID-19. Additionally, the patient reported increased thirst and increased urination a few days prior to presentation. He denied any abdominal pain, nausea, vomiting, vomiting, dizziness, lightheadedness, syncopal episodes, palpitations, or any other symptoms. He also denied any family history of sudden cardiac death. Vital signs on presentation were significant for a temperature of 99.9 degrees Fahrenheit, blood pressure of 120/70 mmHg, heart rate of 106 beats per minute (bpm), respiratory rate of18 breaths per minute, and oxygen saturation of 95% on room air. 

**Figure 1:**
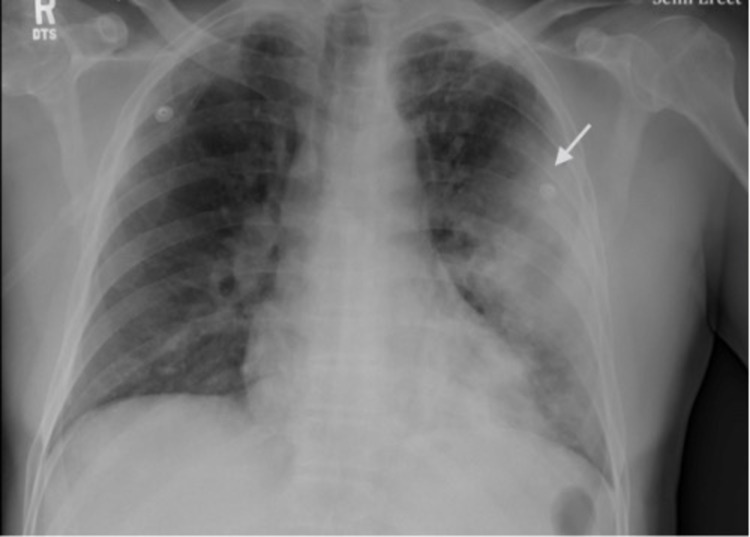
Initial chest x-ray showing large airspace consolidation in the left midlung which has a cavitary appearance. Biapical reticular opacities, left more than right.

**Figure 2:**
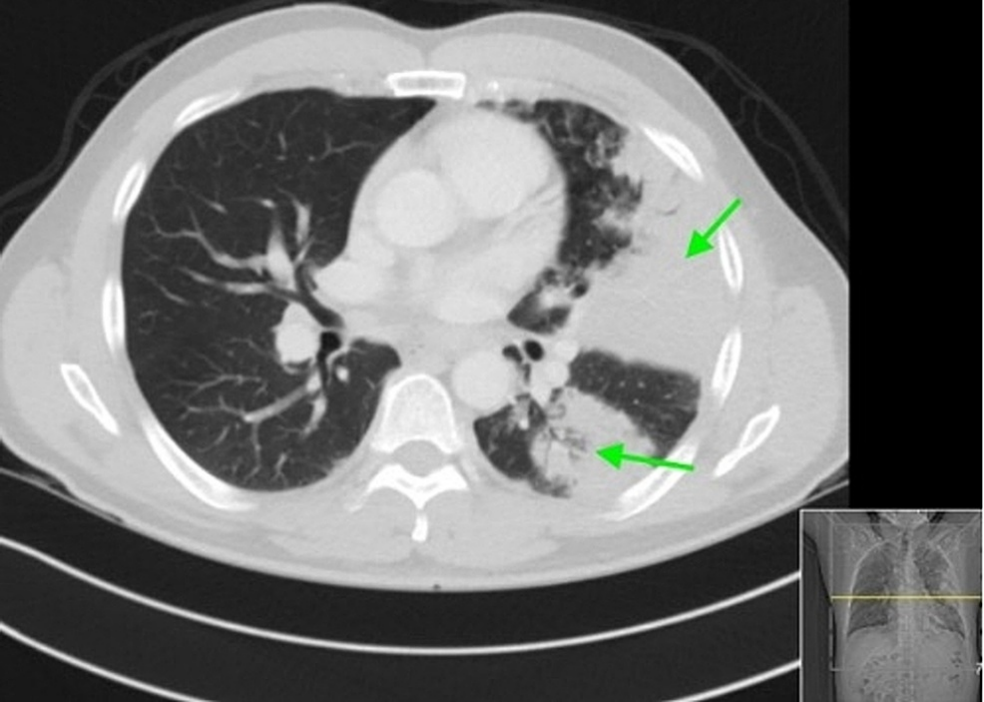
CT showing ground glass airspace opacities bilaterally in combination with dense areas of pulmonary consolidation particularly involving the left upper lobe with the consolidation of at least half of the left upper lobe.

Complete laboratory values on presentation are listed in Table 1. Influenza A and B PCR were negative. The patient tested positive for SARS-CoV-2 by real-time reverse-transcription polymerase chain reaction (rRT-PCR). Chest x-ray (CXR) showed: large airspace consolidation in the left midlung which has a cavitary appearance and biapical reticular opacities left more than the right as seen in Figure 1. Computed tomography (CT) of the chest showed multiple ground glass airspace opacities bilaterally in combination with dense areas of pulmonary consolidation particularly involving the left upper lobe with the consolidation of at least half of the left upper lobe as seen in Figure 2.

**Table 1:** Significant laboratory values upon presentation to the ED

	Reference Range	
White Blood Count (WBC)	4.8-10.8 K/UL	10.3
Hemoglobin	14-18 gm/dL	12.9
Platelet	130 K-400 K	140
Polysa	42%-75%	93.2
Absolute neutrophil count- Polys ABSA	1.4-6.5 K/UL	9.6
Lymphs	20.5-51.1	4.9
Lymphs ABSA	1.2-3.4 K/UL	0.5
Lactic acid	0.5-2.2 mmol/L	1.4
Sodium	136-144 mmol/L	116
Potassium	3.6-5.1 mmol/L	3.8
Creatinine	0.7-1.2 mg/dL	0.81
Osmolality	280-295 mosm/kG	242
Brain Natriuretic Peptide (BNP)	<100 pg/mL	117
Troponin	0.00-0.08	0.03

 

**Figure 3:**
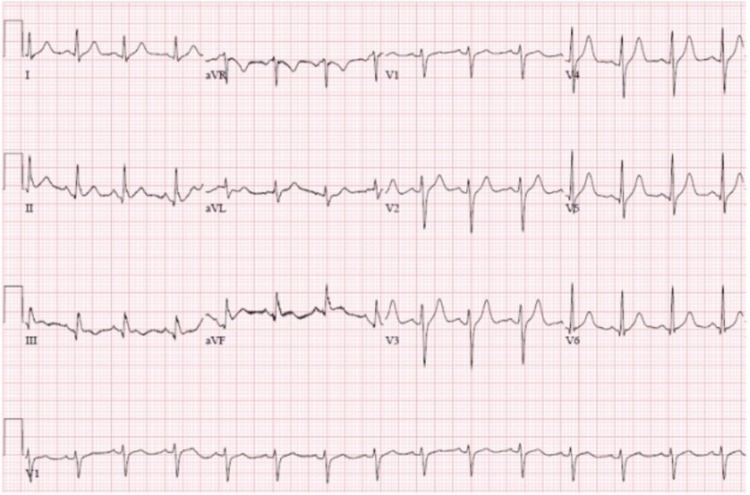
The patient’s EKG with the resolution of fever and correction of electrolytes

An electrocardiogram on presentation demonstrated a coved ST-segment elevation in V1-V2 followed by a negative T-wave, suggesting Brugada pattern type 1 without evidence of ischemic changes (Figure 3). He was initially started on supplemental oxygen via nasal cannula, Tylenol, hydroxychloroquine, azithromycin, ceftriaxone, a regimen that was thought to be advantageous at that time. ECG changes were corrected with the resolution of fever and correction of electrolytes (Figure 3). Throughout the hospital stay, the patient’s oxygenation continued to worsen requiring non-invasive ventilation in the form of bilevel positive airway pressure (BiPAP) and eventually mechanical intubation due to progressively worsening hypoxemic respiratory failure. The hospital course was complicated by severe barotrauma, pneumothorax, hypotension requiring pressor support, atrial flutter, and several complicating bacterial respiratory tract infections. On day 54 of hospitalization, the patient went into a systolic cardiac arrest and despite maximum efforts and initiation of full ACLS protocol, the patient unfortunately died.

## Discussion

Brugada syndrome is an autosomal dominant genetic disorder characterized by a pseudo-right bundle branch block and persistent ST-segment elevation on right-sided pericardial leads on the ECG in conjunction with an increased risk of ventricular tachyarrhythmias and sudden cardiac death. Mutation in the SCN5A gene, which encodes for the alpha subunit of voltage-gated sodium channels is responsible for 30% of all genetically identified Brugada syndrome. In the type 1 Brugada ECG pattern, the elevated ST segment (≥2 mm) descends with an upward convexity to an inverted T-wave, referred to as the “coved type” Brugada pattern. Whereas type 2 Brugada ECG pattern, the ST segment has a “saddleback” ST-T-wave configuration. The elevated ST segment descends toward the baseline, then rises again to an upright or biphasic T-wave [2]. Patients with typical ECG features who are asymptomatic and have no other clinical criteria are said to have the Brugada pattern. The diagnosis of Brugada syndrome is based on clinical criteria and ECG manifestations. Evidence of typical ECG manifestations and the presence of at least one of the following criteria are needed for the diagnosis of Brugada syndrome. These clinical criteria include a family history of sudden cardiac death in a relative younger than 45 years of age or the presence of ECG type 1 in a family member, ventricular tachyarrhythmia, or symptoms associated with arrhythmias such as seizures, nocturnal agonal respiration, or syncope [2].

Multiple factors have been found to trigger or unmask the Brugada pattern. The primary mechanism is the disruption of ionic gradients across myocyte membrane and the dysfunction of the sodium channels. Medications, including antiarrhythmics acting as sodium channel blockers, such as flecainide and propafenone, psychotropic medications, anesthetics such as propofol, and toxins such as cocaine [3]. Fever can also be a trigger for both inductions of Brugada pattern ECG abnormalities and cardiac arrest among persons known to have Brugada pattern ECG or Brugada syndrome. The mechanism is likely due to temperature-induced changes in the action potential and sodium channels [4]. Type 1 Brugada pattern was found to occur 20 times more in febrile patients than patients who are afebrile [5]. Fever was found to be the precipitating factor of cardiac arrest in 18% of Brugada syndrome arrests [6]. Another potential trigger is electrolyte disturbances such as hyperkalemia and hyponatremia [7]; hyponatremia has been reported in the literature as a rare trigger for the Brugada pattern [8]. It is suspected that severe hyponatremia diminishes the electrochemical ionic gradient by decreasing the inward sodium current and leaving the transient outward current unopposed [9]. When dealing with a mechanically ventilated patient who develops a Brugada pattern, an essential point to be aware of is to avoid certain anesthetics such as propofol, which is also known to trigger the Brugada pattern [10].

Brugada pattern has been noted to occur in systemic infections [11], including viral infections such as influenza [12], likely as a result of fever. COVID-19 is a newly recognized infectious disease that has caused a worldwide pandemic, presenting mainly with fever, pneumonia, and hypoxic respiratory failure. Upon performing a literature review in PubMed, we have found similar cases of COVID-19 and Brugada pattern with the resolution of the pattern following defervescence [13,14]. However, one case was reported by Lugenbiel for a COVID-19 patient with a Brugada pattern that persisted despite the resolution of fever [15]. It is now well known that the COVID-19 causes extrapulmonary effects likely secondary to cytokine storm, causing multisystem inflammation, and organ failure. Patients with COVID-19 are at risk of cardiovascular complications, including arrhythmias; [16] whether it is the inflammatory effects on the heart or direct myocardial injury that can unmask Brugada syndrome is yet to be investigated more. Our case report is unique in that this is the first case report to our knowledge where the presence of both fever and hyponatremia in a patient with COVID-19 has triggered Brugada ECG pattern.

Patients with Brugada pattern who are asymptomatic with no history of ventricular tachyarrhythmias or family history of sudden cardiac death have a low risk of arrhythmia or cardiac arrest; therefore, require no treatment apart from the control of provoking factors and monitoring [17]. On the other hand, for patients with Brugada syndrome, those who have survived sudden cardiac arrest, or those with a history of syncope due to ventricular tachyarrhythmias, implantable cardiac defibrillator device placement is the treatment of choice. In patients who refuse ICD or have a contraindication to ICD such as reduced life expectancy, drug therapy with quinidine or amiodarone is indicated [1,2,17]. Our patient had a Brugada pattern likely multifactorial due to fever and hyponatremia in the setting of COVID-19 infection. His EKG normalized after his fever resolved and hyponatremia improved. Unfortunately, he was intubated on mechanical ventilation and developed complications related to COVID-19 infection, and died later on.

## Conclusions

In conclusion, we report a case of a 58-year-old man who presented to the emergency department with complaints of pleuritic chest pain associated with fever and generalized body ache, was positive for COVID-19, labs were significant for hyponatremia and on EKG found to have type 1 (coved) Brugada EKG pattern. Our patient’s EKG normalized once fever and hyponatremia improved. These patients should have serial EKG monitoring and aggressive antipyretic therapy to prevent life-threatening arrhythmia. Our case is unique and adds to the limited number of cases showing association of Brugada pattern EKG with fever and hyponatremia-induced by COVID-19.
